# Medical History from the Earliest Times

**Published:** 1892-11-19

**Authors:** E. T. Withington


### Medical History from the Earliest Times.

By E. T. Withington, M.A., M.B. Oxon.

### XXXII.—ARABIC MEDICINE. (5) HOSPITALS AND INSTITUTIONS.

On the 14th of June, A.D. 918, our old acquaintance Sinan ben Tsabet opened the hospital founded by the Lady Seidet in the Jahya market place at Bagdad. It had an endowment equal to £300 a month, and Sinan was head physician, but he got no pay, and himself subscribed £100 towards another hospital founded in the same year by the Caliph Moktadir. In 925 the Vizier Chakan built a hospital in the Motadhal street, and in 977 the Emir Adad-adaula inaugurated the largest of the Bagdad hospitals, which had a staff of twenty-four medical officers, among whom we find Tsabet ben Sinan and a member of the family of Bachtishua. It is said that the great Rhazes was requested to choose a site for one of these hospitals, and that he hung up pieces of meat in various parts of the city, declaring that the one in which putrefaction last appeared would mark the most suitable position. At least two hospitals were built in the preceding century, one of them by Motawakkel's mother (about 840), while the first Moslem institution for the sick had been founded as early as 707 by the Caliph Welid, who also made special arrangements for the blind, and for lepers. The Arabic medical student had thus ample opportunities for following the advice of Haly Abbas. All the above-mentioned institutions were probably on the model of the Nestorian hospital at Gondisapor, which had existed since the fifth century, and is last heard of about the year 869, when its superintendent, Sabur ben Sahel, published the first known pharmacopœia, which was adopted throughout the Eastern Caliphate.

But the most famous and interesting of Moslem hospitals were those of Damascus and Cairo. About the year 1160 the Emir Nureddin, the Light and Defender of the Faith, having reconquered Edessa and Antioch, and driven back the crusading hosts of Louis VII. and Conrad III., founded, as an appropriate thank-offering to the God of battles, a great hospital at Damascus. "It has never had its like in the world," says Khalil Daheri, and it contained besides departments for diseases of the eye, &c., a medical library and lecture-room, where the tall emaciated figure of Nureddin himself might sometimes be seen among the hearers. The same writer gives us an interesting glimpse of it nearly three centuries later. "While at Damascus in the year 831 (1427) I had with me a certain Persian, a man of wit and intelligence, who followed the rites of the four orthodox sects, performing them all at the same time. When he went over the hospital and saw the patients' diet, and all their comforts and advantages, which are without number, he pretended to be ill and stayed three days there. The physician having felt his pulse recognised his case and prescribed any diet he liked, so he was fed upon young chickens, cakes, sherbet, and all manner of fruits. But after two days the doctor wrote a prescription implying that a guest should not stay beyond the third day. They say there has never been a fire at this hospital since it was built."

Ahmed ben Toulun, the first independent ruler of Egypt, founded, we are told, a hospital there in 874, at a cost of about £30,000. It contained two baths, for men and women respectively, and a department for the insane, and ben Toulun visited it every Friday—the Moslem Sabbath—till a lunatic threw an apple at him, after which he came no more, though he still took an interest in the place. All writers agree in ascribing the first place among Moslem institutions for the sick to the great Mansuri hospital at Cairo. Al-Malek al-Mansur Gilavan, before he became Sultan, was campaigning in Syria against the Greeks, 1276, when he was seized with an attack of colic. Being relieved by medicines from the hospital founded by the "Defender of the Faith" at Damascus, he vowed in his gratitude that he would one day build a yet nobler institution. As soon as he ascended the throne this vow was fulfilled, and such was the zeal of the Sultan and his officials that the hospital, which was begun in June, 1283, was completed in May, 1284. Masons and carpenters were brought from all parts of Egypt; loiterers in the street and passers-by, whatever their rank, were obliged to assist in the holy work, insomuch that "most people avoided going that way;" and the favour of heaven was strikingly manifested by the discovery of two copper caskets filled with gems and gold respectively of sufficient value to defray all the expenses of the building. The hospital was endowed with an income of about £25,000, and contained four great courts, each with a fountain in the centre, wards for each separate disease, a lecture-room, and a department for attending patients at their own homes. It also included an academy, an orphanage, and a chapel, where fifty chaplains recited the Koran day and night without intermission for all who chose to hear. Musicians and story-tellers were provided for the benefit of those troubled with sleeplessness, and the convalescent patient received at his departure five pieces of gold—about fifty shillings—that he might not be obliged to return to work immediately.

With regard to Western hospitals, we have little information except the vague and probably exaggerated assertion that there were fifty such institutions in Cordova, but a fragmentary manuscript has survived, which gives an interesting picture of how medicine was taught among the Arabs in the days of William the Conqueror. Mohammed Ettiminy, a medical student at Toledo about the year 1066, has left us part of a note book containing descriptions of fifty cases treated by his "master," perhaps in the out-patient department of the local hospital. The following is the sixteenth case from the French of Leclerc. "A man came and said he had a swelling on the upper eyelid like a wart. My master told me to go and feel whether it was movable or not. I did so, and it moved like a pea under the skin. When I told him this he bade me evert the eyelid and see if it projected internally. I found nothing. Then he said: 'That is the disease called chalazion; rub it with olive oil, and apply a bread poultice.' The patient did so for some days, and was cured. 'Shall I send him away?' said I to my master. 'Yes, if you know what chalazion is, and how many kinds there are.' 'Three!'" Then the master gives a description of chalazion and its varieties.

If it be true that the Arabs burnt the Alexandrine Library, they certainly did their best to atone for that crime. Even private individuals possessed libraries containing over 100,000 volumes, that of the Mansuri Hospital was so extensive that it required six librarians, and immense collections were accumulated at Bagdad, Bokhara, Cairo, and Cordova. But they have all vanished before the combined destructive energies of savages and theologians. When the Berbers invaded Egypt they carried off the rich bindings of the books, and threw their contents in two heaps, which were covered with sand, and formed conspicuous mounds long known as the "Hills of the Books." When the Mongols destroyed Bagdad, we are told that they used the remains of the libraries to build a bridge over the Tigris, the waters of which great river ran black with ink for many days. In Spain, the Regent al-Mansur, acting on the advice of theologians "and other austere persons," burnt the whole of Hakem's great library, except those books which treated of useful arts or orthodox religion. The Holy office is said to have destroyed at least a million Arabic volumes, and Cardinal Ximenes cast five thousand copies of the Koran into the flames with his own hands. Many medical works, doubtless, perished at this period, and we must especially regret the "Book of Histories of the Bagdad Hospital" and the "Autobiography," both attributed to the great Rhazes.

The decline of the Arabic power was nearly as rapid as its rise. In 1236, St. Ferdinand of Castile took Cordova, and twenty-two years later a more terrible destruction fell upon Bagdad at the hands of the Mongols. That city, indeed, rose again from its ashes, but the town which had formed the cradle of Arabic medicine was swept from the face of the earth, and future travellers sought in vain even for the ruins of Gondisapor.

